# Integrating agent-based models and clustering methods for improving image segmentation

**DOI:** 10.1016/j.heliyon.2024.e40698

**Published:** 2024-11-29

**Authors:** Erik Cuevas, Sonia Jazmín García-De-Lira, Cesar Rodolfo Ascencio-Piña, Marco Pérez-Cisneros, Sabrina Vega

**Affiliations:** Universidad de Guadalajara, CUCEI, Av. Revolución 1500, Guadalajara, Jal, Mexico

**Keywords:** Metaheuristic algorithm, Hybridization, ABM, Agents, Firefly, Image processing, Image segmentation

## Abstract

Image segmentation through clustering is a widely used technique in computer vision that partitions an image into multiple segments by grouping pixels based on feature similarity. Although effective for certain applications, this approach often struggles with the complexity of real-world images, where noise and random variations can significantly affect feature homogeneity, leading to incorrect pixel classifications. To address these limitations, this paper introduces a novel hybrid image segmentation method that combines an agent-based model with a clustering technique to enhance segmentation accuracy and robustness. The method starts with an agent-based model as a preprocessing step aimed at homogenizing pixel intensities within each region. In this model, pixels adjust their intensities based on a consensus reached within their neighborhood, promoting a more uniform feature distribution. Subsequently, the Firefly metaheuristic clustering method is applied to segment the preprocessed image into distinct regions. Metaheuristic techniques, distinguished from classical clustering methods, possess the capability to adaptively navigate through a broad solution space to discover optimal clustering configurations. This adaptability makes them suitable for complex image datasets. The efficacy of the proposed hybrid segmentation method has been tested on various images, employing key quality indices for evaluation. Experimental outcomes demonstrate that this approach yields superior segmented images, showcasing enhanced quality and robustness compared to other segmentation methods.

## 1 Introduction

Image segmentation [1] is a critical step in the field of computer vision, which involves partitioning an image into distinct segments or regions. This process simplifies the representation of an image, making it more manageable and easier to analyze. Image segmentation has numerous applications in various fields, including medical imaging [2], biological sciences [3], autonomous vehicles [4], and augmented reality [5]. Despite the progress made in algorithmic approaches, solving image segmentation problems is still a challenging task due to the variability in image characteristics such as lighting conditions, textures, object shapes and sizes, the presence of noise and occlusions [6].

Several image segmentation methods have been proposed in the literature some examples include the techniques [7,8] such as edge detection, region-based segmentation, thresholding, and machine learning. Edge-based segmentation [9] is particularly notable for its utilization of techniques that identify the boundaries of objects within images through the detection of pixel intensity discontinuities. Prominent among these edge-detection methods are gradient-based techniques, including the Sobel [10], Prewitt [11], Canny [12], and Laplacian of Gaussian [13] operators. Studies comparing edge detection methods have underscored the superior performance of the Canny edge detector, although it's acknowledged that such techniques are prone to the influence of image noise, potentially leading to the creation of misleading edges. Region-based segmentation [14], on the other hand, categorizes an image based on the similarity of pixel characteristics such as intensity, color, and texture, with Region Growing and Split and Merge [15] being exemplary methods. Thresholding strategies delineate objects or areas from the background by establishing a threshold value, with multilevel thresholding facilitating the segmentation of various intensity levels [16]. Various strategies exist for threshold selection, with the Otsu method standing out as a classical approach [17]. Despite their utility, these methods are sensitive to noise and necessitate the identification of an optimal threshold, which may not be universally applicable across different images. Additionally, deep learning techniques [18], notably convolutional neural networks (CNN) [19], represent a modern approach by training models to segment images through the identification of features learned autonomously. Deep learning-based image segmentation methods have achieved remarkable success, yet they come with several notable disadvantages. One of the primary challenges is the need for large, labeled datasets for training, as deep neural networks require extensive amounts of data to learn meaningful features and generalize effectively. Acquiring and annotating such datasets can be time-consuming, costly, and sometimes impractical, especially in specialized domains like medical imaging. Additionally, these methods are computationally intensive, demanding significant hardware resources such as GPUs, which may limit their use.

Other important segmentation approaches use clustering techniques in their operation. Image segmentation methods based on clustering techniques [20] involve partitioning an image into segments or clusters based on the similarity of pixel characteristics. Among the most popular clustering-based segmentation approaches are K-Means clustering [21], Mean Shift [22], and Fuzzy C-means. K-Means clustering is widely used for its simplicity and efficiency. It partitions an image into K clusters, where each pixel is assigned to the cluster with the nearest mean value, resulting in a segmented image where each cluster represents a distinct region. Mean Shift clustering, on the other hand, is a non-parametric technique that iteratively shifts data points towards the mode (the highest density of data points in the region) which can adaptively adjust the size and shape of the window for each cluster, making it robust in identifying complex structures in images. Fuzzy C-means [23] extends the idea of K-Means by allowing pixels to belong to multiple clusters with varying degrees of membership, which results in smoother and more gradual segmentation boundaries, making it particularly useful for images where the transition between regions is not sharply defined. In Ref. [24], a novel affinity matrix to improve membership regularized fuzzy clustering for image segmentation is presented. It combines pixel and region-level information to calculate affinity values and uses fixed cluster centers to reduce noise impact. Experimental results show that this method presents competitive results. Clustering-based image segmentation methods face significant challenges when dealing with noise in pixel data, as these algorithms are inherently sensitive to variations in the feature space. Noise, which typically manifests as random pixel intensity fluctuations, can distort the true distribution of data points, leading to inaccurate estimations of cluster centers. This misrepresentation can result in incorrect pixel assignments, where noisy pixels are grouped into the wrong clusters, thereby producing segments that do not accurately represent the actual content of the image.

Metaheuristic algorithms [25] are other methods used as an alternative to classical clustering methods for image segmentation. Metaheuristic methods are global optimizers methods that offer a versatile approach for clustering [26]. Different from classical clustering methods, metaheuristic techniques can efficiently explore different clustering configurations without being confined to a specific model. Metaheuristic techniques work by iteratively adjusting parameters and evaluating solutions based on a fitness function tailored to the clustering task. One example of such methods is the Firefly Algorithm [27] which is a metaheuristic optimization technique inspired by the flashing behavior of fireflies, which is utilized as a clustering method in various domains, including image segmentation. In the context of clustering, each firefly represents a potential solution, where its position in the search space corresponds to the cluster centers of the dataset. The brightness of a firefly is associated with the fitness of the solution it represents, typically measured by an objective function related to minimize the intra-cluster distance while it is maximized the inter-cluster distance. The Firefly Algorithm's ability to navigate through complex and multimodal landscapes makes it particularly effective for clustering applications, where traditional methods may struggle with the intricacies of real-world data distributions [28,29]. Image segmentation methods based on metaheuristic algorithms, while flexible and capable of navigating complex solution spaces, have several notable disadvantages. One of the most critical aspects is the design of an objective function. Designing an objective function for image segmentation using metaheuristic methods presents several challenges owing to the complex and multifaceted nature of the images. The primary difficulty lies in defining a function that accurately represents the quality of segmentation across diverse images while balancing various factors, such as intra-segment homogeneity, inter-segment separation, and spatial coherence. Real-world images often contain varying textures, overlapping objects, noise, and illumination changes, making it challenging to create a single objective function that adequately accounts for all of these variations.

Although segmentation algorithms often deliver promising results across various applications [26], their effectiveness is notably limited when dealing with complex real-world images [30]. Such images typically contain noise and random variations that can severely alter the distribution of the data points within the feature space [31]. This disruption complicates the accurate classification of pixels, making it difficult to determine whether a pixel belongs to a specific object or background and leading to misestimations. Consequently, pixels affected by noise may be incorrectly assigned to an object to which they do not belong [32], resulting in segments that fail to accurately represent the true content of the image. This misclassification undermines the reliability and accuracy of the segmentation process, thus reducing its effectiveness. The persistent presence of noise and variability in real-world images continues to be a significant challenge for segmentation methods, highlighting the need for more refined and robust techniques that can handle these complexities and improve segmentation accuracy [33].

On the other hand, agent-based modeling (ABM) [34] is a sophisticated simulation technique that is employed to examine the complexities of various systems, encompassing a wide array of scenarios that are centered around autonomous entities or agents, each possessing distinct, predefined characteristics. In a simulated environment, these agents engage with one another and make decisions based on a set of fundamental rules. As the simulation progresses, each agent interacts with its neighbors, and in doing so, modifies its own attributes in response to these interactions. This process of attribute adjustment enables the emergence of intricate, unforeseen global phenomena, thereby showcasing the ability of ABM to generate complex outcomes from simple individual behaviors. The versatility and effectiveness of ABM in replicating and studying dynamic and complex effects have led to its application across multiple fields, including computer science [35] and the social sciences [36], making it an indispensable tool for dissecting and understanding multifaceted systems and behaviors.

This paper introduces a novel hybrid image segmentation technique that integrates agent-based modeling and clustering methods to improve the accuracy and robustness of image segmentation. The method begins with an agent-based model that aims to homogenize pixel intensities across different regions of the image. In this process, individual pixels modify their intensities to align with a consensus derived from their neighboring pixels, creating a more uniform distribution of features in the image. After this preprocessing phase, the segmentation process employs the Firefly metaheuristic clustering method to divide the image into distinct segments. Metaheuristic algorithms are well-suited for exploring large solution spaces and discovering optimal configurations for clustering that classical methods may not find. This combination of agent-based preprocessing and metaheuristic clustering effectively addresses the complexities of diverse image datasets and produces high-quality, robust segmentation results. The proposed method has been extensively validated using various images and quality indices as benchmarks. Experimental results show that the hybrid approach outperforms traditional segmentation methods, producing superior segmented images with enhanced robustness. This represents a significant advancement in the field of image segmentation.

The main contribution of this paper can be summarized in three points:

*Development of a Novel Hybrid Segmentation Method*: The study introduces a new hybrid image segmentation technique that effectively combines an agent-based model with the Firefly metaheuristic clustering algorithm. This integration improves segmentation accuracy by leveraging the strengths of both approaches, offering a more robust solution compared to traditional clustering methods.

*Enhanced Noise Resilience through Preprocessing*: By employing an agent-based model as a preprocessing step, the proposed method homogenizes pixel intensities within regions, reducing noise effects and promoting a more uniform feature distribution. This preprocessing step ensures better segmentation performance, particularly in complex real-world images where noise and random variations often hinder accuracy.

*Adaptability of Metaheuristic Clustering*: The incorporation of the Firefly metaheuristic clustering method allows the hybrid approach to adaptively explore a broad solution space, facilitating optimal clustering configurations. This adaptability enhances segmentation performance on diverse set of images, demonstrating superior results in terms of segmentation quality and robustness compared to other methods.

The remainder of this paper is organized as follows: Section 2 offers an overview of agent-based models, detailing their fundamental principles and relevance to segmentation tasks. Section 3 describes the Firefly algorithm clustering technique, explaining its operation and suitability for image segmentation. Section 4 introduces the proposed hybrid approach, integrating agent-based models with the Firefly algorithm for improved segmentation performance. Section 5 presents the experimental results, demonstrating the effectiveness of the proposed method through various metrics. Section 6 discusses the time analysis, providing insights into the computational efficiency of the approach. Finally, Section 7 concludes the study, summarizing key findings.

## 2 Agent-based models

Agent-based modeling (ABM) [37] is a computational modeling technique that simulates the actions and interactions of autonomous agents (which can be individuals or collective entities such as organizations or groups) with a view to assessing their effects on the system as a whole. It offers a powerful framework for analyzing complex systems where the collective behavior of agents following simple rules can lead to emergent properties that are not always predictable from the rules themselves. Each agent in an ABM is characterized by a set of attributes and behaviors that define its actions within a simulated environment.

The ABM approach comprises four main elements [38]: agents, neighborhoods, environment, and rules. Each of them is described individually below.

### 2.1 Agents

An agent-based model is constructed around a group of M agents $\left\{X_{1},X_{2},\ldots ,X_{M}\right\}$ where each agent i has a specific set of N attributes $\left\{x_{i1},\ldots ,x_{iN}\right\}$ that enable them to tackle problems and exhibit varied behaviors in response to their environment. These agents are governed by a set of rules that dictate their behavior and state, allowing them to either preserve their current state or undergo changes as they interact with other agents within their environment. The dynamics of their movements, interactions, or transitions in state are all dependent on these predetermined behavioral rules and attributes.

In an agent-based model (ABM), an agent is an autonomous, individual entity that operates within a defined environment, interacting with other agents and its surroundings according to a set of simple rules. Each agent possesses distinct characteristics and behaviors, enabling it to make decisions, adapt, and modify its state based on its interactions and observations. The use of agents is crucial for modeling the heterogeneity of complex systems, as each agent can be designed to represent different attributes, roles, or strategies within the system. This capability allows ABMs to capture the diverse behaviors and variations found in real-world systems, where entities often exhibit varying properties, preferences, and reactions. For example, in social systems, agents can represent individuals with distinct goals and social connections, while in biological systems, they can mimic organisms with varying survival strategies and environmental interactions. By allowing agents to interact dynamically and adaptively, ABMs can simulate emergent phenomena—unpredictable patterns that arise from the collective behavior of agents—thereby providing insights into the behavior and evolution of heterogeneous systems that would be difficult to achieve with traditional modeling approaches. This flexibility makes agent-based modeling a powerful tool for studying and understanding the complexity and diversity inherent in many natural, social, and artificial systems.

### 2.2 Environment

The environment refers to the virtual space or context within which agents operate and interact. It serves as a foundational backdrop that defines the spatial and sometimes temporal parameters of the model, dictating how and where agents can move, interact, and be influenced by their surroundings. The environment can range from a simple grid m×n that represents spatial locations where an agent $X_{i,j}$ (i∈1,…,m;j∈1,…,n) can interact, to a complex representation of real-world landscapes, social structures, or abstract spaces, depending on the model's purpose.

In an agent-based model (ABM), the environment is the virtual space or context within which agents operate, interact, and make decisions. It serves as the framework that defines the boundaries, rules, and conditions affecting agents’ behavior. The environment can represent physical spaces, such as geographical areas in ecological models, or abstract spaces, like networks in social simulations. It often contains resources, obstacles, or stimuli that influence agent behavior, acting as both a constraint and an enabler for agent interactions. The relationship between agents and the environment is dynamic and reciprocal—agents can modify the environment through their actions (e.g., consuming resources, altering paths), and the environment, in turn, impacts the agents by providing information, resources, or changing conditions that affect their states and decisions. For instance, in an urban simulation, the environment may represent city infrastructure, with agents interacting by moving through streets, using resources, or responding to traffic signals. This interplay between agents and their environment is fundamental to ABM, as it allows for the emergence of complex patterns and phenomena, reflecting how individual behaviors are shaped by and contribute to the overall system. This interdependence enables ABMs to realistically simulate how entities interact with and adapt to their surroundings, offering valuable insights into the dynamics of real-world complex systems.

### 2.3 Neighborhood

**Fig. 1:**
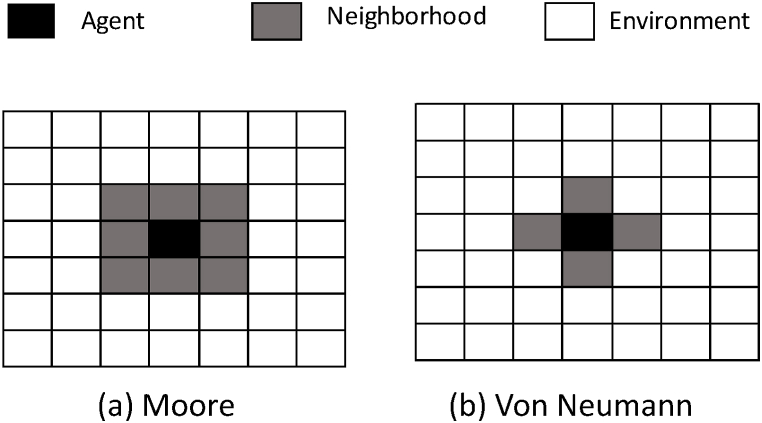
The two types of neighborhoods (a) Moore and (b) Von Neumann.

When agents collaborate within a rectangular array m×n, each agent $X_{i,j}$ have the ability to interact with any element within the array itself, or with agents situated within a specific neighborhood. There are two different types of neighborhoods: Moore and Von Neumann. The Moore neighborhood encompasses both the cardinal (North, South, East, West) and the diagonal neighbors of an agent, resulting in a total of eight surrounding cells in a two-dimensional space. This approach allows an agent to interact with a broader set of neighbors, promoting richer and more diverse interaction dynamics. On the other hand, the Von Neumann neighborhood is more restricted and includes only the four cardinal neighbors of an agent, omitting the diagonals. This results in a cross-shaped pattern of interaction that limits the interactions to four adjacent cells. Each type of neighborhood defines a different scope and scale of interaction, influencing the patterns of spread, diffusion, and the overall behavior of the system. Fig. 1 illustrates the two types of neighborhoods Moore (see Fig. 1(a)) and Von Neumann (see Fig. 1(b)).

In an agent-based model (ABM), the neighborhood refers to the local area around an agent that determines which other agents or environmental elements it can interact with or be influenced by. It defines the scope of an agent's interactions, capturing the agents or cells considered “neighbors”. The type of neighborhood plays a crucial role in shaping the model's dynamics and outcomes. Two common types of neighborhoods are the Von Neumann and Moore neighborhoods, each with distinct characteristics. The choice between these neighborhood types can significantly impact the behavior of the model. For instance, the Moore neighborhood often leads to faster information or influence spread because of its broader reach, resulting in quicker convergence or more pronounced emergent patterns. The Von Neumann neighborhood, on the other hand, can lead to more localized interactions, affecting the diffusion, clustering, or competition dynamics among agents. The selection of the neighborhood type must align with the specific requirements of the system being modeled, as it directly affects how agents perceive their surroundings and make decisions, ultimately influencing the global behavior and emergent phenomena in the simulation.

### 2.4 Rules

Rules in agent-based models (ABMs) serve as the fundamental directives that guide the behavior, interactions, and decision-making processes of agents within the model [39]. These rules are crucial because they encapsulate the logic according to which agents assess their environment, make choices, and change their states or positions, thereby driving the dynamics of the entire system. They can range from simple if-then statements to complex algorithms that incorporate randomness, learning mechanisms, or adaptation over time. The importance of rules lies in their ability to model the heterogeneity of agent behaviors and interactions, which in turn allows for the emergence of complex, system-level phenomena from the bottom-up aggregation of individual actions. This emergent behavior is one of the hallmarks of ABMs, offering insights into how local interactions can lead to unexpected global patterns and trends. By carefully designing these rules, modelers can explore how changes at the micro-level affect the macro-level outcomes, making ABMs a powerful tool for studying systems across a wide range of disciplines, from ecology and economics to social sciences and urban planning. The adaptability and specificity of rules also enable the simulation of real-world scenarios with a high degree of fidelity, allowing researchers and policymakers to test hypotheses, explore scenarios, and predict outcomes in a controlled yet realistic manner.

In an agent-based model (ABM), rules are the predefined behavioral guidelines that dictate how agents interact with one another and their environment. These rules are essential as they define the decision-making processes of agents, guiding their actions, responses, and adaptations over time. Rules determine how agents perceive their surroundings, when and how they react, and how they update their states based on interactions. The outcome of the simulation largely depends on these rules, as they shape the agents' behavior and interactions, ultimately driving the emergence of complex global patterns from individual actions.

## 3 Firefly algorithm clustering method

The Firefly clustering algorithm [40] combines the metaheuristic Firefly method with K-means. This approach is designed to enhance the effectiveness of clustering by optimizing the selection of initial cluster centroids, a critical step in the K-means algorithm that significantly impacts its outcome. This method operates through a sequence of well-defined steps, leveraging the unique advantages of both algorithms.

### 3.1 Initialization phase

In our approach, each firefly represents a potential solution, where a solution in this context refers to a specific set of initial centroids for the clusters. Therefore, during this stage, the centroids assume a random value within the allowed limits. In this phase, the number of clusters k that the algorithm is aimed to identify from the data set is also defined.

### 3.2 Application of the firefly algorithm

The Firefly Algorithm is inspired by the flashing patterns and behavior of fireflies, where each firefly is attracted to others based on their brightness. In this clustering method, the brightness of a firefly is analogous to the quality of the clustering solution it represents, evaluated using the objective function defined in Eq. (1).

(1) $$J\left(C\right)=\sum_{i=1}^{k}\sum_{x\in C_{i}}{\left\|x-\mu_{i}\right\|}^{2}-\alpha \sum_{i=1}^{k-1}\sum_{j=i+1}^{k}{\left\|\mu_{i}-\mu_{j}\right\|}^{2}$$

where C represents the set of clusters $\left\{C_{1},\ldots ,C_{k}\right\}$, k is the number of clusters, $C_{i}$ is the set of data points in cluster i, x is an element of the dataset, $\mu_{i}$ is the centroid of cluster i, ‖∙‖ denotes the Euclidean distance, α is a regularization parameter that balances the importance of intra-cluster compactness versus inter-cluster separation (its nominal value is 0.6). In Eq. (1), the first term of the function aims to minimize the sum of squared distances between each data point and the centroid of its cluster, ensuring that clusters are as tight as possible. The second term, subtracted from the first, seeks to maximize the distances between the centroids of different clusters, ensuring that each cluster is as far apart as possible from others.

During the search strategy of the firefly method, solutions adjust their positions (i.e., the proposed centroids) based on the attractiveness to other fireflies, which diminishes with distance. This mechanism allows the algorithm to search for better solutions by combining local search (moving towards the brighter fireflies) with random exploration (the inherent randomness in the movement), aiming to find the global optimum in the solution space.

### 3.3 Application of the K-means algorithm

Once the Firefly Algorithm has iteratively adjusted the fireflies' positions towards those representing better clustering solutions, these optimized positions of the fireflies are used as the initial centroids for the K-means clustering algorithm. This step is crucial as it sets a more informed starting point for K-means, potentially leading to more accurate clustering outcomes.

With the initial centroids derived from the Firefly Algorithm, the K-means algorithm proceeds through its standard iterative process. This involves assigning each data point in the dataset to the nearest centroid to form clusters and then recalculating the centroids based on the current cluster assignments. This process repeats until the centroids stabilize and no further changes in cluster assignments occur, indicating convergence.

The K-means [41] algorithm clusters data by partitioning it into k groups based on similarity. It operates in three main stages:

1. **Initialize:** Choose k initial centroids.
2. **Assign:** Assign each data point $x_{i}$ to the nearest centroid $\mu_{j}$.
3. **Update:** Recalculate centroids as the mean of all points in each cluster.

These steps repeat until the clusters stabilize or reach a maximum number of iterations. The algorithm minimizes the total within-cluster variance, defined in Eq. (2).

(2) $$J_{K}=\sum_{i=1}^{k}\sum_{x\in C_{j}}{\left\|x_{i}-\mu_{j}\right\|}^{2}$$

where $J_{K}$ is the sum of squared distances between data points $x_{i}$ and their respective cluster centroids $\mu_{j}$, across all k clusters $C_{j}$.

## 4 Proposed algorithm

The proposed methodology comprises two distinct stages, each designed to incrementally enhance the image segmentation process and achieve better accuracy and clarity. The initial stage involves the elimination of noise and unwanted artifacts that could obstruct the precise classification of pixels into their respective clusters. This crucial step ensures that each pixel is evaluated accurately based on its inherent properties, without any distortions produced by random variations. This region homogenization is achieved using an agent-based model. In the subsequent stage, a clustering algorithm is applied that utilizes the Firefly metaheuristic technique. The algorithm, with its ability to navigate complex solution spaces efficiently, classifies each pixel into a group based on the probability that it belongs to a consistent object within the image, based on its intensity level. The end result of this two-stage process is a segmented image where pixels are classified, reflecting a sophisticated understanding and representation of the image's inherent objects and structures.

### 4.1 Phase 1: homogenization of regions using ABM

The model used in the first step employs an agent-based approach that uses a grid with dimensions of m×n elements. Each agent, $X_{i,j}$ (i∈1,…,m;j∈1,…,n) in this array assumes on one of 256 states, which correspond to the various intensity levels present in a grayscale image. At the start of the simulation, the initial condition for all agents is set in such a way that each agent $X_{i,j}$ is given the intensity level of the corresponding pixel $p_{i,j}$ in the same location of the grayscale image I(x,y).

**Fig. 2:**
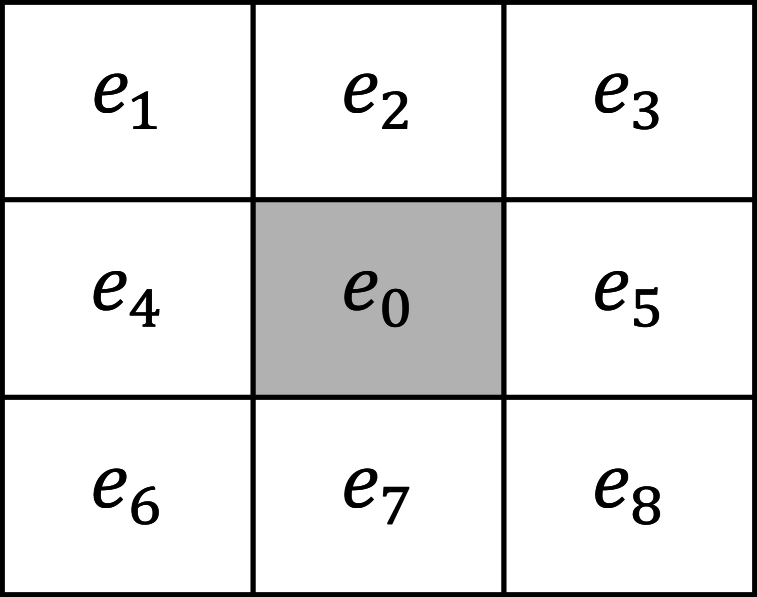
Labeling of the spatial configuration from the relative positions to $e_{0}$ within the grid.

The first phase of the methodology is designed as an iterative process, where a specific number of iterations iter1 are conducted to systematically process each agent within an m×n grid. The selection procedure for each agent adheres to a structured path that begins at the top of the grid and progresses horizontally from right to left, before moving down to the next row and repeating this sequence. To facilitate clarity and simplicity in notation throughout this description, the agent being currently evaluated $X_{i,j}$ in any given iteration t is designated as element $e_{0}$. Surrounding this element, the adjacent agents are identified and referred to as elements $\left\{e_{1},\ldots ,e_{8}\right\}$. This labeling is consistent with the spatial configuration depicted in Fig. 2, which visually outlines the relative positions of the neighbors from $e_{0}$ within the grid.

To calculate how an element $e_{0}$ ($X_{i,j}$) will modify its state in the subsequent iteration t+1, the value $S\left(e_{0}\right)$ that corresponds to the sign of the sum of the signs of the differences among the intensity level of $e_{0}$ and those of the elements within its neighborhood is obtained according to Eq. (3).

(3) $$S\left(e_{0}\right)=\text{sign}\left[\sum_{q=1}^{8}\text{sign}\left(e_{q}\left(t\right)-e_{0}\left(t\right)\right)\right]$$

According to Eq. (1), the variable S is important in determining the relationship between the intensity value of a central agent $e_{0}$ and the intensity values of its neighboring agents. S can assume one of three distinct values, each elucidating a specific condition within the agent's immediate vicinity. When S=1, it signifies that the majority of the neighbors possess intensity values greater than $e_{0}$, indicating that $e_{0}$ is in a relatively darker region compared to its surroundings. Conversely, S=−1 denotes a scenario where most of the neighbors have intensity values that are lower than that $e_{0}$, suggesting that $e_{0}$ is situated in a lighter region. The value S=0 is indicative of two possible scenarios: the first scenario occurs when all neighboring values share the same intensity level as $e_{0}$, where the region already exhibits a uniform intensity level. The second scenario arises when the neighborhood is evenly split, with half having higher intensity values and the other half lower, suggesting that $e_{0}$ is at the boundary between two distinct regions, effectively acting as an edge that delineates a transition from one intensity zone to another.

From the values of S, the agent-based model implements three rules. The first rule $R_{1}$, shown in Eq. (4), considers the scenario where S is equal to 1. In this case, the value of the central element $e_{0}$ must be increased, as most of its neighboring agents have a higher value. This ensures that the region tends to homogenize at each iteration.

(4) $$R_{1}:\mathrm{if}\;S=1\;\mathrm{then}\;e_{0}\left(t+1\right)=e\left(t\right)+\Delta$$

The second rule $R_{2}$, exhibited in Eq. (5), considers the case when S=−1. Under this condition, most of the neighbors to $e_{0}$ have a smaller intensity value than $e_{0}$. Thus, to promote homogeneity it is necessary to decrease the value of $e_{0}$.

(5) $$R_{2}:\mathrm{if}\;S=-1\;\mathrm{then}\;e_{0}\left(t+1\right)=e\left(t\right)-\Delta$$

The third rule $R_{3}$ (see Eq. (6)) addresses the problem when S=0. Under these conditions, either the entire region already has a homogeneous intensity, or the central agent is an edge. In order to maintain these conditions, in both cases it is not necessary to modify the intensity value of $e_{0}$.

(6) $$R_{3}:\mathrm{if}\;S=0\;\mathrm{then}\;e_{0}\left(t+1\right)=e\left(t\right)$$

**Fig. 3:**
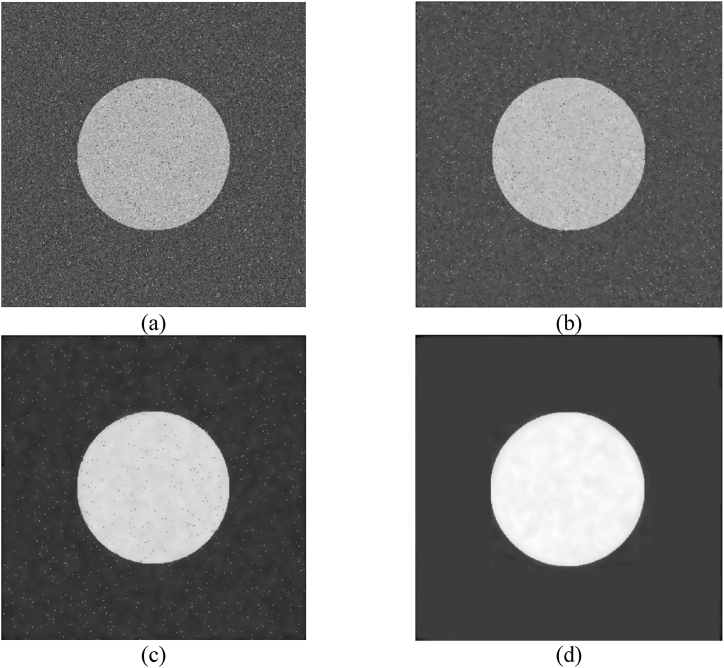
Impact of iterative intensity modifications on an image with the agent-based model. 3(a) original image. Results in 40 (3(b)), 60 (3(c)) and 100 (3(d)) iterations.

Fig. 3 serves as a visual guide to understanding the dynamics of the agent-based model, particularly highlighting how iterative adjustments in pixel intensity can significantly homogenize the image. Initially presented in Fig. 3(a) is the original image, which serves as the input for agent-based model. This image is distinctly divided into two regions, each marked by varying levels of intensity. The intensity values of the first region follow a Gaussian distribution with a mean of 50 and a standard deviation of 20 (N(50,20)), while the second region's intensity values are sampled from a Gaussian distribution with a mean of 200, maintaining the same standard deviation (N(200,20)). The image has also been contaminated with 2000 points of salt and pepper noise. This is 1000 points at random positions with heat of 0 and 1000 with value of 255. The main objective of applying the agent-based model to this image is to achieve intensity homogenization within these regions. Despite the stark initial contrast between them, the algorithm systematically adjusts the intensity values, diminishing the differences and promoting uniformity. This convergence process is illustrated through successive iterations, with Fig. 3(b), (c), and 3(d) capturing the state of the image after 20, 40, and 100 iterations, respectively. These snapshots trace the model's efficacy in gradually homogenizing the regions, illustrating a clear trajectory towards reducing intensity discrepancies and unifying the pixel values within each distinct region, thus demonstrating the practical impact of the model's iterative intensity modification mechanism.

### 4.2 Phase 2: application of the firefly clustering method

After the agent-based model successfully homogenizes the regions within an image, the subsequent phase involves the application of a clustering method. This crucial step is significantly facilitated by the prior homogenization process, as it ensures that the regions within the image are now characterized by uniform intensity levels, effectively reducing the complexity of the clustering task. With the elimination of inhomogeneous intensity levels, the clustering algorithm can more accurately and efficiently assign each pixel to its corresponding class, which represents a specific object or feature within the image. This efficient classification is possible because the homogenization process minimizes the variance within each region, making it easier for the clustering method to identify and group pixels based on their now-consistent characteristics. Consequently, this approach enhances the overall accuracy of the segmentation process, as it directly aids in the clear delineation of objects within the image, ensuring that each pixel is correctly classified into its appropriate category based on its attributes.

During this particular phase of the segmentation process, the clustering method employs the firefly algorithm, well-known for its efficiency in solving optimization problems. This method is configurated considering two key parameters: the number of clusters (k) and the number of iterations (iter2). The parameter k is crucial as it directly influences the segmentation outcome by determining the number of distinct classes or categories within the image, essentially dictating the level of detail and differentiation the algorithm aims to achieve in the segmentation. On the other hand, the iterations parameter sets the breadth of the search within the solution space, indicating how thoroughly the algorithm will explore potential solutions before arriving at the most optimal configuration. This exploration is driven by the goal of optimizing a specific objective function, as detailed in Eq. (1), which aims to enhance the clustering quality by minimizing intra-cluster variance while maximizing inter-cluster distinction. Through this process, the firefly algorithm iteratively adjusts the clustering configuration, navigating towards an optimal solution that accurately reflects the inherent structure and distinctions within the image data, thereby facilitating a precise and meaningful segmentation based on the defined objective.

Once the group g (g∈1,…,k) to which each pixel $p_{i,j}$ belongs has been obtained, a new image is generated in which at each position of the pixel $p_{i,j}$ the group g is placed in place of its intensity. This image corresponds to the segmented image.

### 4.3 Computational procedure

After presenting the two key stages of the segmentation algorithm in detail, the subsequent section considers a comprehensive examination of the algorithm as a whole. This thorough analysis is facilitated through the presentation of Algorithm 1, which is depicted in the form of pseudocode to offer a clear and systematic overview of the computational steps involved. Algorithm 1 is carefully structured into discrete lines, each of which corresponds to a specific operation within the overall procedure.

The algorithm initiates (line 1) its process by establishing the values for two critical parameters: iter1 and iter2. These parameters are instrumental in governing the iteration count for the two main components of the algorithm—the agent-based model and the firefly-based clustering algorithm, respectively. The setting of iter1 dictates the extent to which the agent-based model will operate to homogenize the intensity levels across the image's regions, thereby preparing the image for the subsequent clustering phase. Similarly, iter2 determines the depth of exploration the firefly algorithm will undertake in search of the optimal clustering configuration, ensuring a thorough examination of the solution space. Additionally, the algorithm specifies the magnitude of intensity adjustment Δ (increment or decrement) that the agent-based model employs to gradually unify the pixel intensity values within each region, a crucial step that significantly influences the pace at which the image achieves uniformity in intensity. The final setup step involves defining the number of clusters k, which directly corresponds to the number of distinct elements or categories into which the image will be segmented. This parameter is pivotal, as it shapes the granularity of the segmentation outcome, thereby determining the level of detail and differentiation captured in the final segmented image.

At the beginning of the procedure, the algorithm considers the original image I as its input, setting the stage for the agent-based model to start its operation. The primary task of this model is to methodically reduce the random variations inherent in the image, often introduced during the capturing process. This reduction is systematically achieved over a predefined number of iterations, denoted by the parameter iter1. As the agent-based model progresses through these iterations, it diligently works towards generating an intermediate output $I_{H}$ characterized by regions of homogeneity. This step is pivotal as it prepares the groundwork for the subsequent phase of the algorithm. Following the generation of $I_{H}$, the clustering method based on the firefly technique is applied. The firefly-based clustering method explore the solution space to identify the most effective clustering arrangement, guided by the objective of optimizing the function described in Equation (1). This optimization process involves a series of iterations, specified by the parameter iter2. The final operation is the production of the segmented image $I_{S}$ that contains the various objects or regions within the original image.

**Algorithm 1:** Pseudocode of the proposed method.

1:	Input: I,k,iter1,iter2,Δ
2:	$I_{H}\leftarrow$ Agent-based-model (I,iter1,Δ)
3:	$I_{S}\leftarrow$ FireflyClustering ($I_{H},k,iter2$)
4:	Output: $I_{S}$

**Fig. 4:**
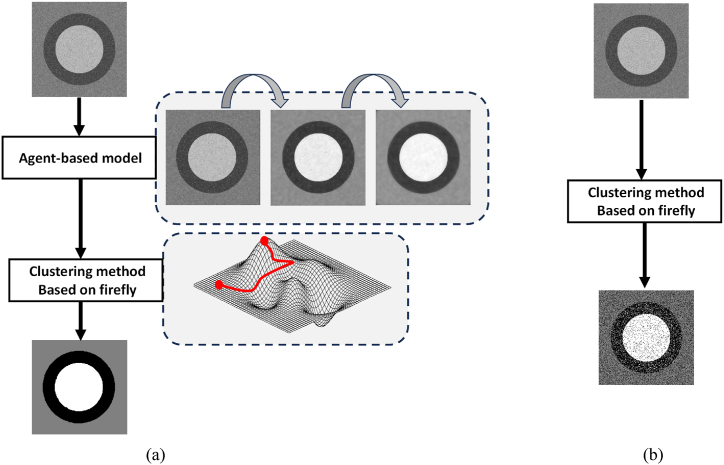
Flow diagrams of the proposed approach (a) and the simple clustering method (b).

The effectiveness of the segmentation process is demonstrated through an illustrative example, which offers a direct comparison of outcomes against those generated by a more straightforward clustering approach. This comparison is visually reinforced by two flowcharts, in Fig. 4(a) delineating the process flow for the proposed method, and Fig. 4(b) outlining the workflow for a simple clustering method. Our proposed method incorporates the integration of an agent-based model with a clustering technique rooted in the firefly algorithm. In contrast, the simple method depends solely on the application of a clustering technique without the preliminary effect provided by the agent-based model. In this comparative scenario, both methods are applied to the same image, which is characterized by three distinct regions. These regions are marked by a notable lack of homogeneity in their intensity levels, exhibiting a standard deviation of 30, and further complicated by the presence of salt and pepper noise, a common challenge in image processing tasks. The flow of operations within both techniques is meticulously documented, providing insight into the step-by-step processing that each method undertakes.

The visual analysis presented in Fig. 4 demonstrates the superior performance of the method that integrates both the agent-based model and the clustering process, based on the firefly technique, in terms of segmentation capabilities. This comprehensive approach effectively identifies and segments the objects within the image. In contrast, the method that relies solely on the firefly technique-based clustering struggles to achieve a clear distinction among the objects in the image. This limitation can be primarily attributed to its inadequate addressal of the random variations in intensity values that characterize the image. These variations introduce complexity that hinders the effectiveness of the simple clustering process in isolating and identifying the distinct objects contained within the image. As a result, the comparative analysis highlights the significance of incorporating an agent-based model as a preparatory step in the segmentation process, showcasing its critical role in enhancing the robustness and accuracy of the segmentation outcome against the backdrop of challenging image characteristics.

## 5 Experimental results

This section presents a comprehensive analysis of the results achieved through the application of our proposed approach, referred to as ABM-FF. To assess its effectiveness and performance, ABM-FF was compared with four well-established segmentation methods widely recognized in the literature. These benchmark methods include the Regularized Fuzzy Clustering (RFC) [24], the Fuzzy C-means-new algorithm [23], a clustering-based strategy, and Otsu's method [17], a classical technique in the domain of image segmentation. This comparative evaluation aims to provide an integral view of how ABM-FF measures up against these traditional and contemporary segmentation methods. By examining a wide range of techniques, from classical approaches like Otsu's method to more modern strategies such as fuzzy C-means, this analysis highlights the advantages and capabilities of the ABM-FF approach and provides valuable insights into its potential in handling diverse and complex segmentation tasks.

To provide a fair and impartial comparison of the segmentation algorithms, we carefully configured each method included in our study according to the recommended parameter settings outlined in their respective references in the literature. This approach ensures that each algorithm is optimized for performance, thereby enabling a reliable evaluation of their effectiveness and efficiency.

**Fig. 5:**
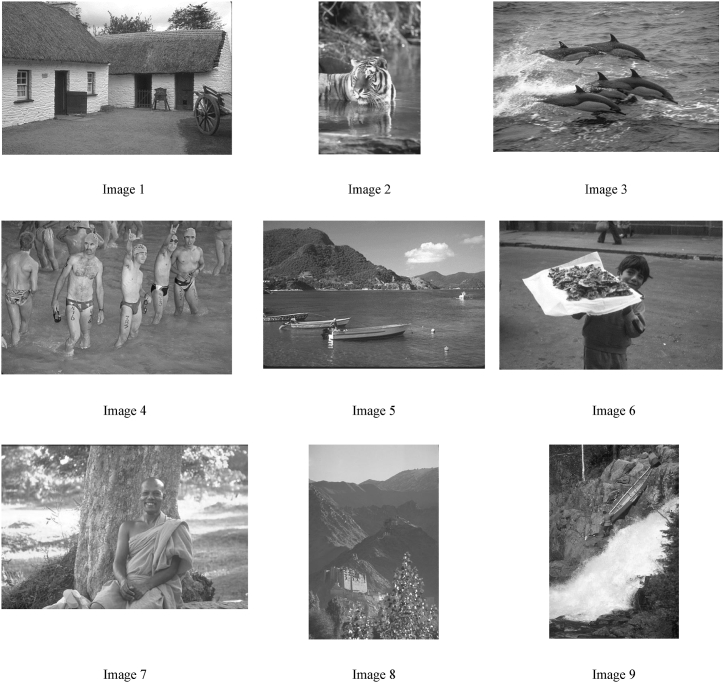
Images used in the experiments.

To numerically assess the effectiveness of our proposed ABM-FF segmentation methodology, we conducted an evaluation using the complete set of 300 images from the Berkeley Segmentation Dataset and Benchmark (BSDS300), a well-known public database for image segmentation. This comprehensive analysis enabled a broad comparison of our approach with established segmentation methods across a diverse range of images. However, due to the space constraints, only 9 images were selected from the dataset for a detailed visual evaluation of the results. These images were chosen to represent challenging scenarios, providing a thorough test of the algorithm's performance under complex conditions. The selected images are presented in Fig. 5, showcasing the segmentation outcomes and highlighting the robustness of the proposed method.

To conduct a numerical assessment of the image segmentation results, our study incorporated a comprehensive set of established quality indices, providing an objective measure of performance [42]. Specifically, we utilized a range of metrics known for their reliability in evaluating the quality of image processing outcomes. These included the Peak Signal-to-Noise Ratio (PSNR), the Structural Similarity Index Method (SSIM), and the Feature Similarity Index Method (FSIM). Each of these indices offers a different perspective on image quality: PSNR measures the ratio of the maximum possible power of a signal to the power of corrupting noise, SSIM evaluates the visual impact of three characteristics of an image (luminance, contrast, and structure), and FSIM considers feature-based similarity between two images. The choice of these metrics was driven by their demonstrated ability to quantitatively and objectively assess the quality of segmented images. These indices were chosen to ensure consistency and comparability with prior studies, as they are widely used in most segmentation research reported in the literature. By employing this methodical approach, our study ensures that the evaluations are both comprehensive and reliable, enabling informed conclusions about the effectiveness of the image segmentation methodologies.

PSNR assesses the level of noise or distortion present in a segmented image by comparing it to the unaltered original image. A greater PSNR value suggests a lower level of distortion or error, implying that the segmented image closely resembles the original image. The PSNR is determined with equations (7), (8)).

(7) $$PSNR=20\;\log_{10}\left(\frac{255}{RMSE}\right)$$

(8) $$RMSE=\sqrt{\frac{\sum_{i=1}^{ro}\sum_{j=1}^{co}\left(I_{Gr}\left(i,j\right)-I_{th}\left(i,j\right)\right)}{M∙N}}$$

where M and N refer the number of rows and columns for the image, respectively. SSIM evaluates how well the segmented image retains the essential structural information present in the reference image. A higher SSIM score indicates a closer resemblance between the segmented and reference images, indicating better segmentation quality. The SSIM is determined by using equations (9), (10)).

(9) $$SSIM\;\left(I_{Gr},I_{th}\right)=\frac{\left(2\mu_{I_{Gr}}\mu_{I_{th}}+C1\right)\left(2\sigma_{I_{Gr}}\sigma_{I_{th}}+C2\right)}{\left(\mu_{I_{Gr}}^{2}+\mu_{I_{th}}^{2}+C1\right)\;\left(\sigma_{I_{Gr}}^{2}+\sigma_{I_{th}}^{2}+C2\right)}$$

(10) $$\sigma_{I_{0}I_{Gr}}=\frac{1}{N-1}\sum_{i=1}^{N}\left(I_{Gr_{i}}+\mu_{Gr}\right)\left(I_{{th}_{i}}+\mu_{I_{th}}\right)$$

where $\mu_{I_{Gr}}$ and $\mu_{I_{th}}$ are the mean values of the original and segmented images, respectively, for each image. The values of $\sigma_{I_{Gr}}$ and $\sigma_{I_{th}}$ correspond to the standard deviation. Constants C1 and C2 are used to avoid instability when $\mu_{I_{Gr}}^{2}+\mu_{I_{th}}^{2}\approx 0$. In this instance, the values of C1 and C2 are experimentally determined to be 0.065.

The FSIM evaluates the structural and textural features of a segmented image by evaluating its similarity to a reference image. A higher FSIM score indicates a greater likeness between the segmented image and the reference image, suggesting superior segmentation quality in terms of preserving important visual features and textures. The FSIM score is calculated by using equations (11), (12), (13), (14), (15)).

(11) $$FSIM=\frac{\sum_{w\in \Omega }S_{L}\left(w\right)PC_{m}\left(w\right)}{\sum_{w\in \Omega }PC_{m}\left(w\right)}$$

(12) $$S_{L}\left(w\right)=S_{PC}\left(w\right)S_{G}\left(w\right)$$

(13) $$S_{G}\left(w\right)=\frac{2G_{1}\left(w\right)G_{2}\left(w\right)+T_{2}}{G_{1}^{2}\left(w\right)+G_{2}^{2}\left(w\right)+T_{2}}$$

(14) $$G=\sqrt{G_{x}^{2}+G_{y}^{2}}$$

(15) $$PC\left(w\right)=\frac{E\left(w\right)}{\left(\varepsilon +\sum_{n}A_{n}\left(w\right)\right)}$$

where Ω refers to the image domain.

The experimental section of this study has been systematically structured into two distinct parts, each designed to examine different aspects of the segmentation methods under evaluation. In the first part, as detailed in section 5.1, we conduct a comparative analysis of the segmentation results derived from the original images displayed in Fig. 5. This evaluation focuses on assessing the visual consistency and numerical indices of the segmented images. Such an approach allows us to comprehensively compare both the perceptual and numerical quality of each segmentation method when applied to unaltered images. This part of the study provides insights into how each method performs in ideal conditions, offering a baseline for their effectiveness. In the second part, outlined in section 5.2, our analysis shifts towards a qualitative and quantitative evaluation of the segmentation methods under conditions of noise. Here, we consider two prevalent types of noise: Gaussian noise, which adds statistical noise having a normal distribution, and salt and pepper noise, which introduces sharp, sudden disturbances in the image data. These types of noise are chosen due to their common occurrence and significant impact on image quality, making them ideal for testing the robustness of segmentation methods. This part of the experimental analysis is essential, as it allows us to objectively determine the resilience of each segmentation method against common distortions and adverse acquisition conditions, thereby assessing their utility in more challenging and realistic scenarios.

### 5.1 Segmentation results over original images

In this subsection, we conduct a detailed analysis of the results obtained by each algorithm featured in our comparative study, focusing on the original images as depicted in Fig. 5. The primary aim of this analysis is to evaluate the capacity of the segmentation methods to effectively delineate and separate the objects within these images. This examination encompasses both visual and numerical comparisons to determine if the methods can consistently and meaningfully segment objects, especially under challenging conditions such as varied textures or poor lighting in the images.

**Fig. 6:**
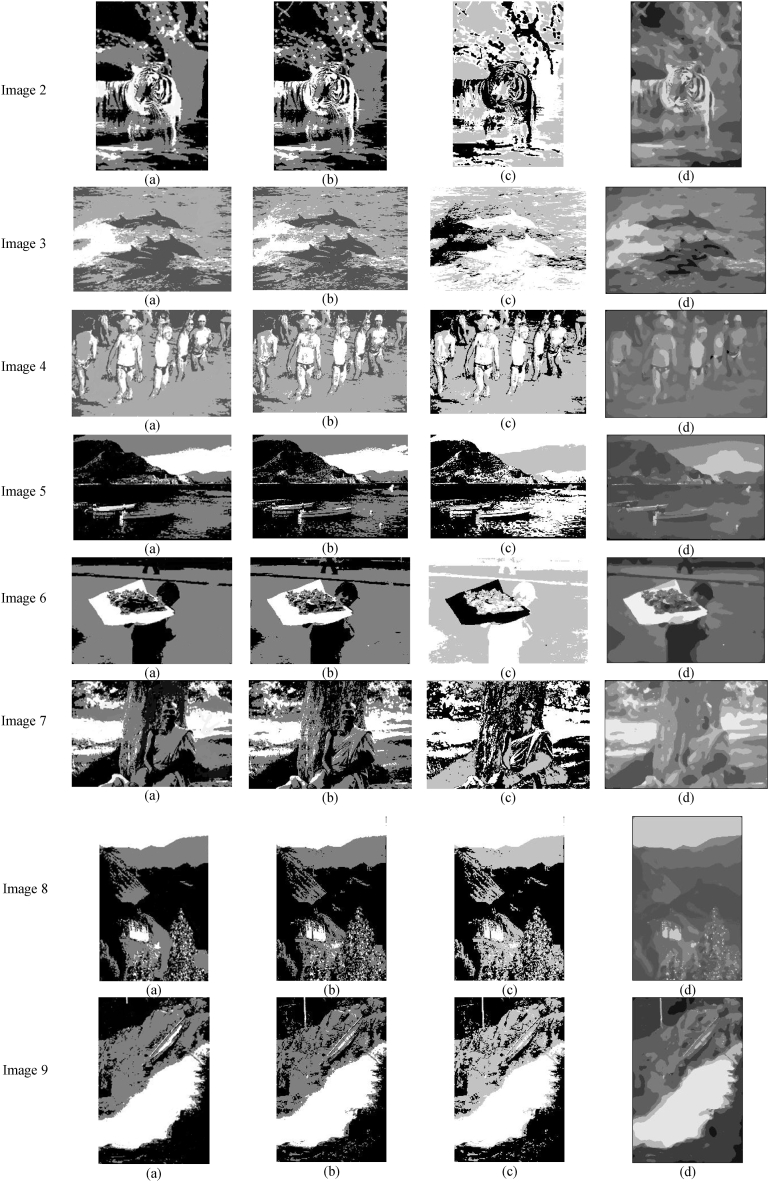
Visual results produced by the four algorithms on the nine images used in our experiments.

Fig. 6 presents the visual outcomes produced by the four algorithms on the nine images used in our experiments. This presentation facilitates a direct assessment of the segmentation results, enabling a clear visualization of how well each method performs in identifying and outlining distinct objects and regions within the images. The comparative insights gained from Fig. 6 are instrumental in understanding the strengths and limitations of each segmentation approach, providing a comprehensive view of their effectiveness in real-world scenarios where image quality may be compromised. This analysis not only highlights the technical capabilities of the segmentation methods but also their practical applicability in handling complex image conditions.

Fig. 6 offers a visual representation of the results from the segmentation of the original images shown in Fig. 5, providing a clear comparison of the effectiveness of different segmentation methods. Notably, the ABM-FF method emerges as the superior technique in this analysis. This method consistently produces well-defined segments that are closely aligned with the actual objects within the images, underscoring its semantic accuracy. In contrast, the methods based on the Fuzzy C-Means-New and the Regularized Fuzzy Clustering (RFC) algorithms struggle with the simultaneous treatment of all regions, particularly in handling textured objects and artifacts, which significantly impairs its performance. Similarly, the Otsu methods exhibit limitations, particularly in their ability to manage the complexity introduced by unwanted pixel variations, resulting in less satisfactory outcomes compared to the ABM-FF method.

The visual evaluation in Fig. 6 distinctly highlights the superior performance of the ABM-FF method in accurately segmenting the images. The robustness of the ABM-FF method is largely attributable to its capability to effectively homogenize the regions within the segmented images. This homogenization ensures that the regions are not only consistent but also semantically coherent, which is essential for an accurate representation of the objects depicted. By maintaining a high level of internal consistency and semantic integrity within these regions, the clustering method based on the Firefly algorithm precisely classifies pixels, facilitating accurate and faithful delineation of objects. The inherent ability of the ABM-FF method to create coherent and semantically meaningful regions significantly enhances the quality of segmentation, enabling it to outperform other methods. Ultimately, the strength of the ABM-FF method in region homogenization stands out as a key factor in its competitive advantage across various image segmentation tasks.

Table 1 provides a comprehensive comparison of the numerical results obtained from applying four different segmentation algorithms to all 300 images from the BSD300 dataset. The table presents average values for each evaluation index calculated by the algorithms across the dataset. An analysis of Table 1 shows that the proposed ABM-FF algorithm consistently outperforms the others across multiple metrics. In terms of the Peak Signal-to-Noise Ratio (PSNR), the ABM-FF achieves higher scores, indicating lower distortion and fewer errors, resulting in a more accurate representation of the original image after segmentation. The algorithm Regularized Fuzzy Clustering (RFC) presents the best value in the Structural Similarity Index Method (SSIM), which measures the preservation of critical structural details that closely match the reference image, highlighting its superior segmentation quality. Although the RFC algorithm obtains a better value in terms of the SSIM index, the proposed method is very close to this value with a similar value. Furthermore, the ABM-FF leads in the Feature Similarity Index Method (FSIM), demonstrating its capability to maintain important visual features and textures, thus emphasizing its advanced segmentation capabilities. These results confirm the effectiveness and reliability of the ABM-FF in image segmentation. In contrast, the Fuzzy C-Means-New and Regularized Fuzzy Clustering (RFC) methods rank second, showing strong segmentation performance but still trailing the ABM-FF in overall accuracy. The Otsu method ranks third, providing reasonable results but falling behind the more advanced RFC, Fuzzy C-Means-New, and ABM-FF algorithms. This ranking highlights not only the superior performance of the ABM-FF but also the distinctive strengths and limitations of each method, clearly demonstrating the unique advantages of the proposed approach in image segmentation.

**Table 1:** Comparison of the numerical averaged results obtained from applying four different segmentation algorithms to all 300 images from the BSD300 dataset.

	RFCM	Fuzzy C-means-New	Otsu	ABM-FF
PSNR	18.1235	17.5439	16.8823	**21.5128**
SSIM	**0.7921**	0.5720	0.4031	0.7894
FSIM	0.6024	0.6321	0.5822	**0.8890**

### 5.2 Performance of segmentation algorithms in the presence of noise

In this subsection, we consider the performance analysis of the four distinct segmentation algorithms—RFC, Fuzzy C-Means-New, Otsu, and the proposed ABM-FF method—specifically focusing on their ability to handle images contaminated with noise. The types of noise considered in these experiments are Gaussian and salt-and-pepper noise, which are frequently encountered in image processing due to common variations and imperfections in the image capturing process. To create a controlled and reproducible testing environment, random noise is artificially introduced to the original images shown in Fig. 5. This approach allows us to simulate the challenges typically faced in real-world scenarios where images may suffer from such distortions. The inclusion of these specific noise types provides a robust framework for assessing how well each algorithm can perform under adverse conditions, thereby offering insights into their effectiveness and reliability in noisy environments. Through this analysis, we aim to identify which segmentation methods are best equipped to maintain high performance and accuracy despite the presence of noise, thereby providing valuable information on their practical applicability in varied and challenging segmentation tasks.

#### 5.2.1 Gaussian noise

**Fig. 7:**
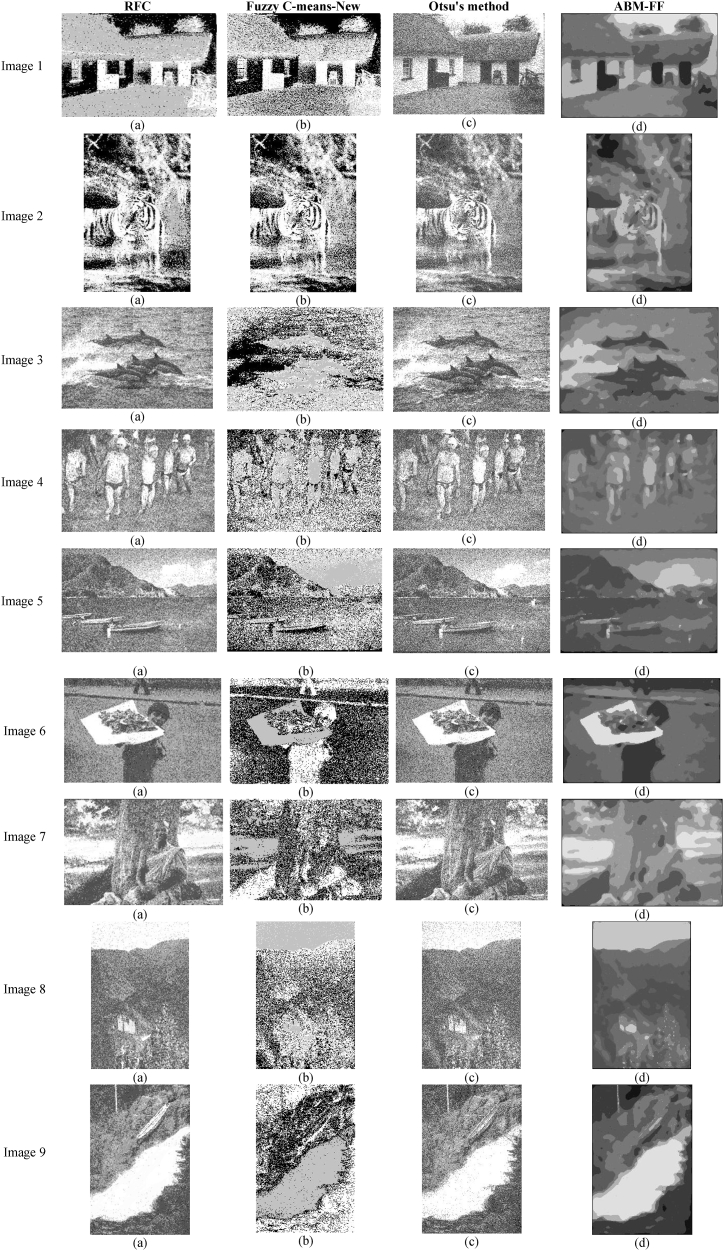
Segmentation results produced by the algorithms considering Gaussian noise.

In this experiment, we specifically focus on the behavior of the segmentation algorithms when processing images subjected to Gaussian noise conditions. To simulate this scenario, Gaussian noise is added to each pixel $p_{i,j}$ in the images, according to the model $p_{i,j}=p_{i,j}+N\left(0,1\right)$ where *N* denotes a normally distributed number with zero mean and a standard deviation of 1. This method of noise addition is chosen to mimic common real-world disturbances that can occur during the image capture process, particularly in lower light conditions or with less sophisticated imaging equipment. The introduction of this type of noise helps to test the robustness of the segmentation algorithms under conditions that challenge their ability to accurately delineate image regions. Fig. 7 presents the outcomes of this noisy input as segmented by the various methods. By examining these results, we can observe and analyze how each algorithm copes with the added complexity of Gaussian noise, providing insights into their effectiveness and adaptability in handling images with such statistical noise distortions.

Fig. 7 presents an analysis of the resilience of different segmentation algorithms when faced with Gaussian noise. The results indicate varying levels of effectiveness among the methods tested. The proposed ABM-FF algorithm demonstrates strong robustness, as it maintains clear and precise segmentation boundaries despite the presence of noise. It consistently delineates objects without noticeable degradation in performance, highlighting its ability to handle noisy conditions effectively. In contrast, the Otsu method performs poorly under the same conditions, with noise significantly blurring object boundaries and leading to inaccurate segmentations. The Fuzzy C-means-New method exhibits the worst performance, struggling to accurately classify and assign pixels due to its sensitivity to intensity changes introduced by noise. This results in a high rate of misclassification, with many object pixels mistakenly labeled as background. The RFC method shows better noise resilience than the Otsu and Fuzzy C-means-New methods but still fails to eliminate noise effectively, leaving many noisy pixels incorrectly classified. The superior performance of the ABM-FF algorithm in handling noisy conditions underscores the importance of developing segmentation techniques that can ensure high accuracy and reliability even in challenging environments, making them suitable for real-world image analysis and processing.

Table 2presents the averaged numerical results from segmenting 300 Gaussian noisy images from the BSD300 dataset using four different algorithms. The data clearly show that the proposed ABM-FF method consistently outperforms the others, achieving the highest scores across the PSNR, SSIM, and FSIM performance indices. Although the ABM-FF's scores are slightly lower than those obtained in noise-free conditions, it still maintains a strong lead over the other methods in noisy scenarios. The RFC algorithm ranks second in all indices, demonstrating greater robustness to noise compared to the Otsu and Fuzzy C-Means-New methods. The Otsu method secures third place, displaying moderate noise resilience, though not as robust as RFC. In contrast, the performance of the Fuzzy C-Means-New method is significantly affected by Gaussian noise, making it the least effective among the tested algorithms. Both the Otsu and Fuzzy C-Means-New methods show notable declines in performance compared to their noise-free results, highlighting their vulnerability to noise-induced distortions. This stark contrast emphasizes the superior design of the ABM-FF, which effectively manages noise while maintaining high segmentation accuracy, even in challenging conditions.

**Table 2:** Averaged numerical results from segmenting 300 Gaussian noisy images from the BSD300 dataset using four different algorithms.

	RFCM	Fuzzy C-means-New	Otsu	ABM-FF
PSNR	17.8712	14.1025	15.6121	**20.9874**
SSIM	0.5524	0.3521	0.3897	**0.6527**
FSIM	0.6000	0.4678	0.5729	**0.7257**

The good performance of the proposed ABM-FF approach can be attributed to its sophisticated mechanism for homogenizing regions that contain pixels with intensity variations. This capability is largely a result of the rules implemented by the agent-based model, which are particularly adept at smoothing out variations within a region while carefully preserving the edges of objects. This process effectively mitigates the impact of added noise by adjusting the intensity of noisy pixels, enabling them to converge towards the intensity values of their neighboring pixels within the same region. This strategic modulation of pixel values ensures that the noise does not disrupt the inherent structure and clarity of the image. Once the regions within the image are homogenized, segmenting the image into distinct objects becomes significantly more straightforward. The uniformity in the intensity of regions means that the pixels can be grouped into their respective objects without the variations that typically lead to misclassification. This not only enhances the accuracy of the segmentation process but also the reliability of the results, as each pixel is more likely to be correctly classified according to its true object. Therefore, the ABM-FF method not only excels in dealing with noise but also in creating a clear and accurate representation of different objects within an image, showcasing its robustness and effectiveness in image segmentation under challenging conditions.

#### 5.2.2 Salt-and-pepper noise

**Fig. 8:**
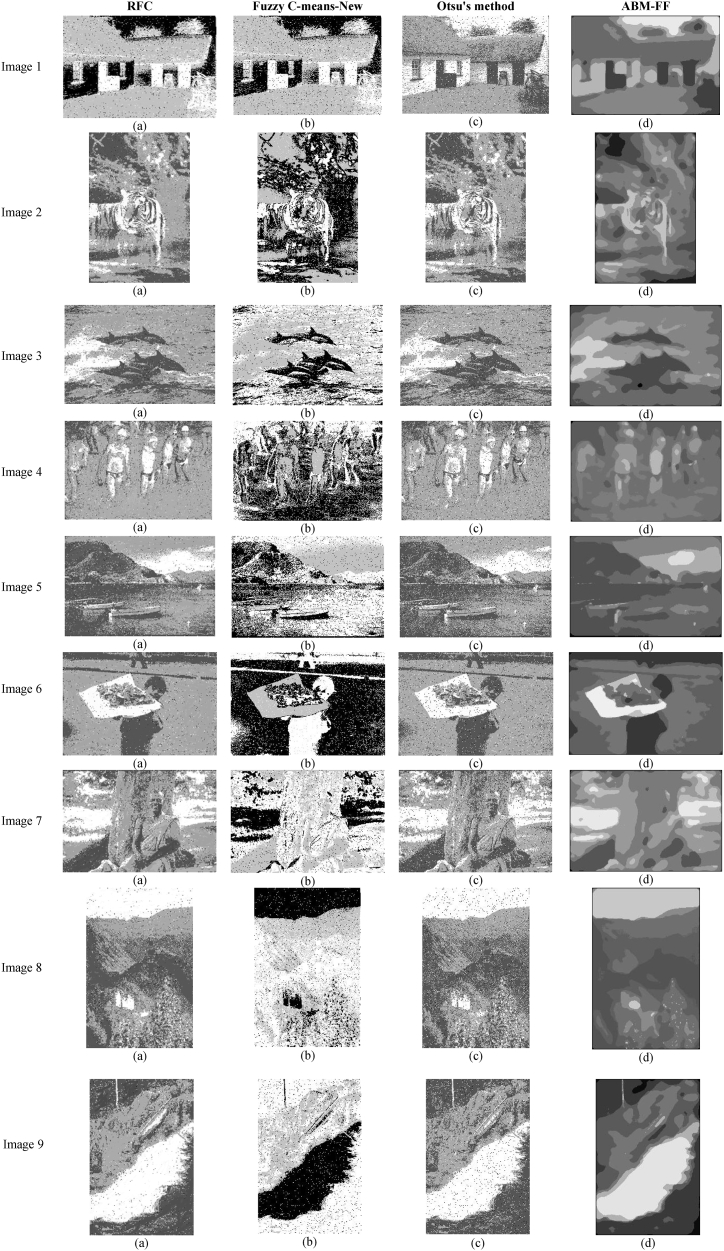
Segmentation results produced by the algorithms considering salt-and-pepper noise.

In this experiment, we examine how segmentation algorithms perform when processing images afflicted with salt-and-pepper noise. This type of noise is characterized by the random addition of two extreme pixel values, zero and 255, which represent the darkest and brightest possible intensities, respectively. To simulate this condition, these values are randomly integrated into the image at a density *d* of 0.1. This density implies the number of pixels Np in the image (Np=M∙N∙d) that are altered to either zero or 255. Salt-and-pepper noise is a common issue in real-world scenarios, often resulting from transmission errors, sensor defects in cameras, or the degradation of photographic materials. The introduction of such noise presents a significant challenge for segmentation algorithms, testing their capability to accurately and effectively delineate regions within the image despite these disruptions. This experiment aims to highlight the resilience and adaptability of different segmentation methods under conditions that closely mimic these challenging disturbances. Fig. 8 presents the outcomes of applying these methods to the noisily altered images, providing a visual representation of how each algorithm copes with the task of segmenting under the influence of salt-and-pepper noise.

Fig. 8 presents an analysis of the robustness of different segmentation algorithms when faced with Gaussian noise. The results indicate varying levels of effectiveness among the methods tested. The proposed ABM-FF algorithm demonstrates strong robustness, as it maintains clear and precise segmentation boundaries despite the presence of noise. It consistently delineates objects without noticeable degradation in performance, highlighting its ability to handle noisy conditions effectively. In contrast, the Otsu method performs poorly under the same conditions, with noise significantly blurring object boundaries and leading to inaccurate segmentations. The Fuzzy C-means-New method exhibits the worst performance, struggling to accurately classify and assign pixels due to its sensitivity to intensity changes introduced by noise. This results in a high rate of misclassification, with many object pixels mistakenly labeled as background. The RFC method shows better noise resilience than the Otsu and Fuzzy C-means-New methods but still fails to eliminate noise effectively, leaving many noisy pixels incorrectly classified. The superior performance of the ABM-FF algorithm in handling noisy conditions underscores the importance of developing segmentation techniques that can ensure high accuracy and reliability even in challenging environments, making them suitable for real-world image analysis and processing.

Table 3presents the averaged numerical results in terms of PSNR, SSIM, and FSIM indices following the segmentation of images affected by salt-and-pepper noise, across 300 images from the BSD300 dataset using four different segmentation methods. The results here reflect a similar pattern to those observed under Gaussian noise conditions. The proposed ABM-FF algorithm once again excels, achieving the highest scores across all indices, indicating its superior capacity to preserve image quality and integrity despite the presence of significant noise. The RFC algorithm ranks second in all indices, demonstrating better noise robustness compared to the Otsu and Fuzzy C-Means-New methods. While the Otsu method cannot match the performance of ABM-FF or RFC, it still secures third place, showing reasonable results even under the challenging effects of salt-and-pepper noise. In contrast, the Fuzzy C-Means-New method continues to underperform, producing the lowest scores among the tested algorithms. Its consistent struggles with both Gaussian and salt-and-pepper noise highlight its limitations in handling the complexity and randomness introduced by such noise types. Overall, these results reinforce the robustness and efficiency of the ABM-FF algorithm, which consistently leads in maintaining high segmentation performance across noisy conditions, outperforming the other methods tested.

**Table 3:** Averaged numerical results in terms of PSNR, SSIM, and FSIM indices following the segmentation of images affected by salt-and-pepper noise, across 300 images from the BSD300 dataset using four different segmentation methods.

	RFCM	Fuzzy C-means-New	Otsu	ABM-FF
PSNR	17.2147	14.4578	15.0757	**20.9973**
SSIM	0.4787	0.3087	0.3574	**0.5397**
FSIM	0.5978	0.4987	0.4890	**0.7002**

Despite the commendable performance of the proposed ABM-FF algorithm, it is not without its limitations, as evidenced by observations from Fig. 7, Fig. 8. The inherent design of the agent-based model within ABM-FF aims to homogenize pixel intensities within regions to counteract the presence of noise. This strategy, while effective in reducing noise, can inadvertently lead to the merging of neighboring regions that should distinctively stand apart. As a result, this aggressive homogenization can cause a loss of critical image details, with distinct regions blending into one another, thereby diminishing the granularity and clarity of the image. This tendency to oversimplify the image's complexity by merging adjacent regions manifests as a blurring effect, which can obscure important features and textures necessary for accurate and meaningful image interpretation. Therefore, while the ABM-FF algorithm excels in noise reduction, this strength also poses a challenge in maintaining the precise delineation and individual integrity of each region within the image.

## 6 Time analysis

This section expands the analysis by evaluating the performance of the proposed ABM-FF algorithm in comparison with other segmentation methods, focusing specifically on the computational time required by each approach. By examining the time taken for each algorithm to complete the segmentation process, this analysis offers valuable insights into both the efficiency and effectiveness of the methods. Understanding these trade-offs between computational cost and segmentation accuracy is crucial, as some algorithms may provide highly accurate results but at the expense of longer processing times, which may not be suitable for time-sensitive applications. Conversely, faster algorithms may offer reduced accuracy. This discussion highlights the balance that must be struck between achieving precise segmentation and maintaining acceptable computational performance, which is essential for practical implementation in real-world scenarios where both speed and accuracy are important considerations.

**Fig. 9:**
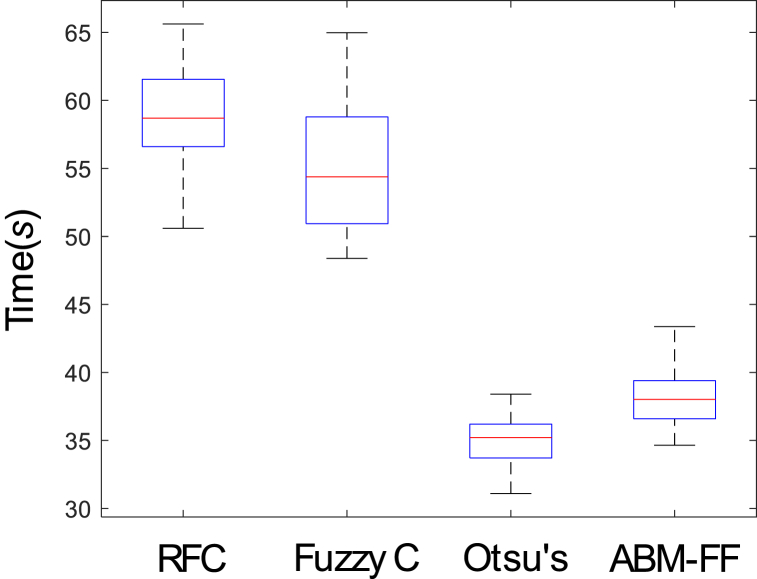
Distribution of the inverted time values for all 300 images from the BSDS300 dataset across the different segmentation techniques.

Segmentation techniques employ a variety of processes, ranging from simple to highly complex, each with distinct computational demands. However, the presence of stochastic components and intricate structures within these methods makes traditional complexity analysis unsuitable for evaluating the algorithms in this study. To assess the efficiency of each segmentation method, an experiment was conducted where each algorithm was run on every image in the dataset. The time taken to produce the segmentation, measured in seconds, was recorded for each run. Fig. 9 presents the distribution of the inverted time values for all 300 images from the BSDS300 dataset across the different segmentation techniques. By using inverted time values, the figure provides a clearer comparison of computational performance, highlighting how quickly each method arrives at a solution. This analysis allows for a direct comparison of the computational efficiency of each segmentation algorithm, showcasing the differences in processing speed and performance across various approaches, and helping to identify the methods that offer a more favorable balance between computational cost and segmentation speed.

An analysis of Fig. 9 shows that Otsu's method achieves the shortest processing times, ranging between 33 and 36 s, indicating that it utilizes the simplest and fastest processing mechanisms among the evaluated algorithms. The ABM-FF method follows closely, with processing times between 37 and 39 s, making it the second-fastest method in terms of computational efficiency. In contrast, the Fuzzy C-means-New and RFC methods demonstrate the longest processing times, exceeding 50 s, highlighting their more complex and slower processing structures. Despite its slightly longer processing time compared to Otsu's method, the ABM-FF method achieves superior results in terms of segmentation accuracy and robustness to noise. Conversely, while Otsu's method is faster, it delivers the lowest quality in terms of segmentation performance. This analysis suggests that the ABM-FF method offers the best balance between segmentation quality and processing speed among all the algorithms examined in this study, making it the most effective compromise for achieving both efficiency and accuracy.

## 7 Conclusions

This paper presents a new image segmentation approach that incorporates agent-based modeling and clustering methods to enhance the accuracy and resilience of image segmentation. The approach commences with an agent-based model that aims to standardize pixel intensities across different regions of the image. In this process, individual pixels modify their intensities to conform to a consensus derived from neighboring pixels, resulting in a more uniform distribution of features in the image. Following this preprocessing step, the segmentation process utilizes the Firefly metaheuristic clustering technique to partition the image into distinct segments. The integration of agent-based preprocessing and metaheuristic clustering effectively tackles the intricacies of diverse image datasets and produces high-quality, robust segmentation outcomes.

To determine the effectiveness and efficiency of the proposed ABM-FF method, it was compared to four widely recognized segmentation methods that have been well-established in the literature. These methods include the Regularized Fuzzy Clustering (RFC), the Fuzzy C-means-new algorithm, two clustering-based strategies, and Otsu's method, a classical technique in the field of image segmentation.

The four segmentation methods were evaluated using both qualitative (visual inspection) and quantitative approaches, specifically focusing on well-established metrics such as the Peak Signal-to-Noise Ratio (PSNR), the Structural Similarity Index Method (SSIM), and the Feature Similarity Index Method (FSIM). These metrics provide a comprehensive view of the performance of each method by evaluating aspects like image fidelity, structural integrity, and feature preservation in the segmented outputs. The computational experiments conducted demonstrate that the proposed method consistently yields superior segmented images. It excels in enhancing the quality and robustness of the segmentation, outperforming other methods across all evaluated indices. The enhanced performance of the proposed method is evident not only through higher numerical scores on these metrics but also through visual assessments, where the segmented images display clearer boundaries, reduced noise, and more accurate representation of the original scenes. This dual approach to evaluation highlights the effectiveness of the proposed method in producing high-quality, robust segmentation results, making it a preferable choice in scenarios demanding high precision and reliability.

Although the ABM-FF algorithm demonstrates impressive capabilities, it also has certain limitations. The agent-based model within the ABM-FF was designed to homogenize pixel intensities within regions, effectively reducing noise. However, this strategy can sometimes lead to the unintended merging of adjacent regions, which should remain distinct. This aggressive homogenization can result in the blending of critical image details, causing distinct regions to lose their clarity and reducing the overall granularity of the segmentation. To address this issue, future work could focus on developing an adaptive system that selectively homogenizes only those regions that semantically represent objects while preserving the texture and detail of regions that should remain separate. This approach would aim to maintain image details while still managing noise effectively, improving segmentation accuracy and visual quality.

## CRediT authorship contribution statement

**Erik Cuevas:** Writing – original draft, Methodology, Conceptualization. **Sonia Jazmín García-De-Lira:** Software, Formal analysis. **Cesar Rodolfo Ascencio-Piña:** Writing – review & editing, Validation. **Marco Pérez-Cisneros:** Writing – review & editing, Resources, Funding acquisition. **Sabrina Vega:** Visualization, Project administration, Investigation.

## Data availability statement

Data will be made available on request.

## Declaration of competing interest

The authors declare the following financial interests/personal relationships which may be considered as potential competing interests:Erik Cuevas is AE in Heliyon If there are other authors, they declare that they have no known competing financial interests or personal relationships that could have appeared to influence the work reported in this paper.
